# Bear baiting risks and mitigations: An assessment using expert opinion analyses

**DOI:** 10.1371/journal.pone.0312192

**Published:** 2024-11-25

**Authors:** Diana J. R. Lafferty, Sarah M. Trujillo, Grant V. Hilderbrand, Andee Sears, Peter Christian, David Payer, Mary Hake

**Affiliations:** 1 Department of Biology, Wildlife Ecology and Conservation Science Lab, Northern Michigan University, Marquette, MI, United States of America; 2 Department of Biology and Wildlife, University of Alaska, Fairbanks, AK, United States of America; 3 National Park Service–Alaska Region, Anchorage, AK, United States of America; Universidade Federal de Mato Grosso do Sul, BRAZIL

## Abstract

Bear baiting is authorized in 12 states, 2 territories, and 8 provinces across North America. In Alaska, more than 70% of lands managed by the National Park Service (NPS) are open to some form of hunting including National Preserves where non-conflicting state wildlife hunting regulations apply. Alaska state regulations authorize bear baiting with few restrictions on the type or amount of bait that can be used to attract bears; although, restrictions related to bait station distance from roads and trails (¼ mile) and cabins/dwellings (1 mile) apply. However, National Preserves host diverse recreational activities in addition to hunting (e.g., hiking, camping, fishing). Because road and trail access to and within Alaska National Park and Preserve lands is limited, hunting and non-hunting-related activities often occur in the same areas–increasing potential for conflict between potentially non-compatible activities. We developed questionnaires about potential impacts on NPS lands, which were distributed to 14 NPS and 27 non-NPS bear research and management experts. We collated respondents’ opinions regarding consistency of bear baiting practices with state and federal mandates for wildlife management. While minor differences in expert opinions were noted, findings from this study are unequivocal. Bear baiting is functionally equivalent to feeding bears, bears may defend a bait station similar to how they would defend a carcass, and bear baiting can lead to human food-conditioning in bears. Bear baiting also increases the likelihood bears will be killed in defense of life and property, and alters natural bear behaviors and ecological processes. Further, current mitigation strategies to minimize public safety risks and potential property damage are inadequate. For example, because bears are known to defend food resources, avoiding food conditioning of bears is central to the educational messaging of all entities that manage bears. In short, bear baiting is a harvest practice that challenges harmony between State mandates, which emphasize hunter opportunity, and NPS mandates that include public safety and natural processes.

## Introduction

State and federal natural resource agencies tasked with managing wildlife resources have differing legal mandates that shape policies and objectives. As an example, Alaska Department of Fish and Game (ADFG) has an obligation to manage wildlife harvest, wildlife populations, and hunters; whereas the National Park Service (NPS) has an obligation for wildlife stewardship that includes safeguarding natural ecosystem processes such as ensuring wildlife behavior is unaltered by human activities, while providing a myriad visitor services, ensuring public safety, and providing adequate facility management. Wildlife harvest is an authorized activity on 70 percent of the areas managed by NPS in Alaska and non-conflicting State of Alaska harvest regulations govern sport harvest on National Preserves. Thus, the NPS strives to manage wildlife harvest opportunities in harmony with broader NPS mandates. Challenge arises when determining if State regulations conflict with NPS mandates. Bear baiting, for example, is harvest practice that continues to challenge the harmony between State mandates, which emphasize hunter opportunity, and NPS mandates that include public safety and natural processes, which are core to the NPS mission.

Alaska hunting regulations require bait stations be at least ¼ mile from any road or trail and at least 1 mile from any dwelling (Bear Baiting: Rules & Requirements - Hunter Education, Alaska Department of Fish and Game [1]), and an individual can register and maintain multiple bait stations in the same general area. Common items provisioned as bear bait include processed and ultra-processed foods such as dog food, bread and pastries, syrup, icing, and bacon grease, as well as natural items including ungulate entrails and carcasses left over from hunting and trapping. Bait station maintenance requires repeated access by road, trail, or aircraft. Though there is a paucity of literature regarding the direct and indirect impacts of bear baiting relative to required wildlife resource management mandates, tension with NPS policy goals is apparent. By allowing bear baiting, the NPS’ ability to meet federal mandates related to safeguarding natural ecosystem processes (Sections 4.4.1 and 4.4.2 of the 2006 NPS Management Policies [2]) is threatened because bear baiting alters bear behavior resulting in increased bear mortality [3], consumption of processed and ultra-processed foods affect bear diet [4] and gut health (e.g., gut microbiome [5]), and the spatial distribution of human-provisioned foods affects space use [6, 7], the diet [8] and gut health [9, 10] of diverse species. For example, by examining camera trap imagery from bear bait sites in Michigan, Chandler et al. (2019) identified >15 species of non-target mammals (e.g., American marten [*Martes americana*], Gray wolf [*Canis lupus*], Moose [*Alces alces*], Snowshoe hare [*Lepus americanus*]) as well as multiple avian species (e.g., Common raven [*Corvus corvus*], Wild turkey [*Meleagris gallopavo*], Turkey vulture [*cathartes aura*]) consuming bear bait that consisted of processed and ultra-processed human foods [9]. In northern Wisconsin, bait contributed to >40% of black bear diets and the authors noted that several non-target species were regularly found at bear bait stations [4]. Moreover, food conditioned bears are more likely to be killed in defense of life or property [11–14] and, even in areas open to bear harvest, non-harvest mortality of bears is viewed as wasteful and something all agencies strive to minimize. Perhaps more importantly, bear baiting jeopardizes the ability of the NPS to provide for public enjoyment activities in a manner that mitigates known risks to public safety because food-conditioned bears pose a greater risk to humans than non-food conditioned bears and may defend baits stations in a manner similar to defense of a carcass [3]. Thus, while the probability or likelihood of a human being mauled near a bait station is small, when it occurs, the event is likely catastrophic. NPS policy states, “The saving of human life will take precedence over all other management actions as the Park Service strives to protect human life and provide for injury-free visits.” (Section 8.2.5.1 Visitor Safety of the 2006 NPS Management Policies).

Because experimentally designed studies to characterize and quantify risks to humans and bears associated with bear baiting are largely impractical as well as ethically nonviable, the literature examining linkages among bear baiting and state and federal wildlife management is scant. Despite the paucity of insight from designed research methodologies, management agencies in areas where bear baiting is an ongoing practice must make decisions if bear baiting will be allowed; and, if so, appropriate seasons, type of bait allowed, and mitigation strategies to reduce potential risks to humans and other non-target species. The NPS in Alaska embarked on public-informed regulatory processes in 2015 and 2020 to address certain hunting practices on preserves and each effort suffered from lack of consensus on the potential impacts of bear baiting. The 2015 rule found bear baiting incompatible with the overall NPS mission, statutes and regulations and the 2020 rule reversed this finding. In 2022, NPS was directed to re-visit the topic of bear baiting. Lacking adequate empirical data, we engaged technical experts as a robust resource to inform management decisions (e.g. [15]). Thus, our objective was to collate expert opinion regarding the impact that bear baiting on NPS lands may have relative to meeting state and federal mandates associated with wildlife and visitor management.

## Methods

To garner diverse expert insight regarding the potential impact of bear baiting on NPS lands relative to simultaneously meeting both state and federal mandates associated with wildlife management, the NPS used a questionnaire with five questions (Table 1) to query 14 NPS technical experts, who, on average had 20.6 years (range 8 to 35 years) of natural resource management and research experience at the time of the query. This 5-question questionnaire was available to NPS technical experts from 15 November through 20 December, 2022. In addition, the NPS used questionnaires including unstructured questions (Table 2) to query 27 non-NPS North American bear management and research biologists from state and provincial agencies, universities, and non-NPS federal agencies, who, on average had 25 years (range 10 to 48 years) of professional bear experience at the time of the query. Further, the majority of the non-NPS individuals queried serve on the International Union for the Conservation of Nature’s North American Bear Expert Team (NABET). This 19-question questionnaire was available to non-NPS technical experts from 28 November 2022 January through 18 January 2023. Additionally, all recipients of the questionnaires were selected based on experience or recommendations from NABET members to ensure geographic and affiliation coverage.

**Table 1:** 5-question questionnaire used to query 14 natural resource and research technical experts from the National Park Service.

1. How many years of professional-level experience do you have as a natural-resource biologist or manager?
2. Is bear baiting a management issue at your park, i.e., does baiting occur on or near your park? yes/no
3. Do you think bear baiting creates a potential public safety risk? If yes, please rate the risk 1–5, with 1 being very low and 5 very high risk.
4. Do you think bear baiting can contribute to an increase in Defense of Life and Property take of bears? yes/no
5. Do you think baiting can alter natural processes or behaviors of bears and other wildlife? yes/no. Elaborate if you wish.

**Table 2:** 19-question questionnaire used to query 27 bear management and research biologists from federal (non-National Park Service), state and provincial agencies, and universities across North America. Each question allowed for one response: strongly agree, agree, neutral, disagree, strongly disagree.

1. Baiting bears using large quantities (e.g., 100s of lbs.) of dog food, pastries, syrup, or other processed foods is functionally equivalent to feeding bears.
2. Baiting bears using large quantities (e.g., 100s of lbs.) of dog food, pastries, syrup, or other processed foods results in a fixed resource that may be defended by a bear in a manner equivalent to how it would defend a carcass.
3. Baiting bears using large quantities (e.g., 100s of lbs.) of dog food, pastries, syrup, or other processed foods may alter natural bear behavior.
4. Baiting bears using large quantities (e.g., 100s of lbs.) of dog food, pastries, syrup, or other processed foods may lead to bears associating food with people.
5. Baiting bears using large quantities (e.g., 100s of lbs.) of dog food, pastries, syrup, or other processed foods may contribute to an increase in the killing of bears (e.g., in defense of life or property).
6. Baiting bears using large quantities (e.g., 100s of lbs.) of dog food, pastries, syrup, or other processed foods has the potential to impact bear behavior beyond the immediate baiting season.
7. Baiting bears using large quantities (e.g., 100s of lbs.) of dog food, pastries, syrup, or other processed foods has the potential to impact other components of the ecosystem beyond bears (i.e., non-target species).
8. Baiting bears using large quantities of natural food items such as ungulate carcasses, salmon, or berries that are added to the landscape or moved from their original location is functionally equivalent to feeding bears.
9. Baiting bears using large quantities of natural food items such as ungulate carcasses, salmon, or berries that are added to the landscape or moved from their original location results in a fixed resource that may be defended by a bear in a manner equivalent to how it would defend a carcass.
10. Baiting bears using large quantities of natural food items such as ungulate carcasses, salmon, or berries that are added to the landscape or moved from their original location may alter natural bear behavior.
11. Baiting bears using large quantities of natural food items such as ungulate carcasses, salmon, or berries that are added to the landscape or moved from their original location may lead to human food-conditioning of bears.
12. Baiting bears using large quantities of natural food items such as ungulate carcasses, salmon, or berries that are added to the landscape or moved from their original location may contribute to an increase in the killing of bears (e.g., in defense of life and property).
13. Baiting bears using large quantities of natural food items such as ungulate carcasses, salmon, or berries that are added to the landscape or moved from their original location has the potential to impact bear behavior beyond the immediate baiting season.
14. Baiting bears using large quantities of natural food items such as ungulate carcasses, salmon, or berries that are added to the landscape or moved from their original location has the potential to impact other components of the ecosystem beyond bears (i.e., non-target species).
15. Stipulations that bait stations are at least ¼ mile from a trail or road resolves any public safety concerns relative to bears defending a bait station.
16. Stipulations that bait stations are at least 1 mile from a cabin or other swelling resolves any public safety or property damage concerns due to bears associating food with people.
17. How many years have you worked in the field of bear management or research?
18. Please provide any additional comments you have on the subject of bear baiting.
19. Would you prefer to be named in the acknowledgments of any publications or summaries of this survey or remain anonymous?

### Ethics statement

As a federal agency, NPS does not have an Institutional Review Board. As required, however, author GVH consulted with the Office Management and Budget (OMB) that oversees public surveys and questionnaires. Importantly, surveys and questionnaires of federal employees are exempt from OMB review. The criteria for review by OMB requires 10 or more non-federally employed respondents to an identical survey. Because this study included unstructured questions and less than 10 non-federal employees/respondents to an identical survey, this study was exempt from OMB review. The university personnel (DJRL, SMT) were not involved in designing the questionnaire or determining the pool of potential respondents and were invited to synthesize survey results that were returned to NPS and to lead manuscript writing. Thus, the study also was exempt from university Institutional Review Board review. Further, questionnaire completion was voluntary, several of the questions were unstructured (see S1 and S2 Tables), individual responses were not tied respondents, and respondents were anonymous but given the option to be acknowledged as a participant via written consent. Thus, by completing the questionnaires, respondents provided written informed consent. However, to ensure anonymity, we redacted geographic identifying information (e.g., [State 1] instead of listing the state referenced. See de-identified supplementary S1 and S2 Tables for respondent response data without restriction.

## Results

### NPS technical experts

**Fig 1:**
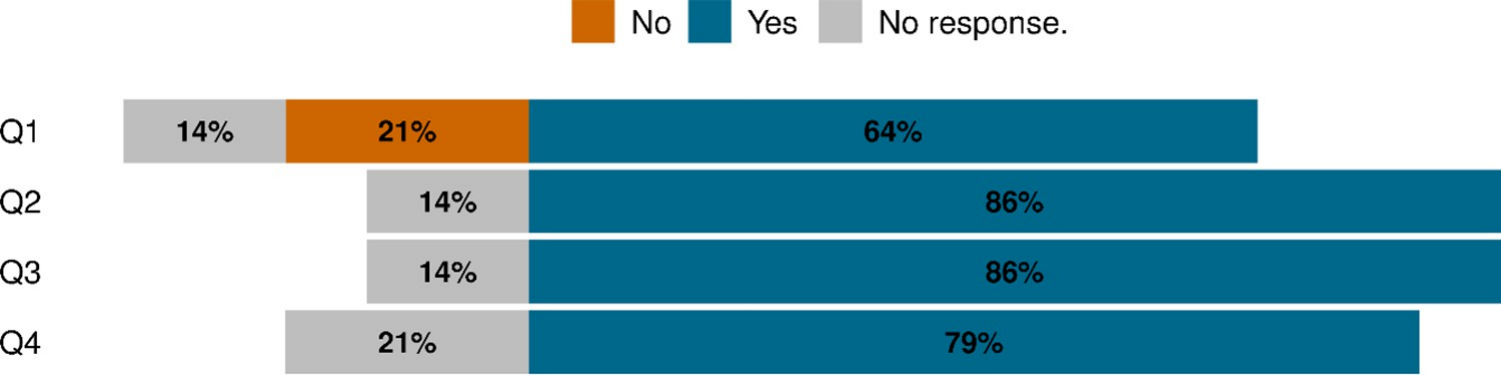
Summary results from the 5-question questionnaire used to query 14 natural resource and research technical experts from the National Park Service.

The 14 NPS technical experts unanimously responded to the query that bear baiting will increase the likelihood of defense of life and property kills of bears and will alter the natural processes and behaviors of bears and other wildlife. In considering the potential for significant human injury or even death, these NPS technical experts considered the overall risk of bear baiting to the visiting public to be moderate to high (Fig 1).

### Non-NPS North American bear management and research biologists

**Fig 2:**
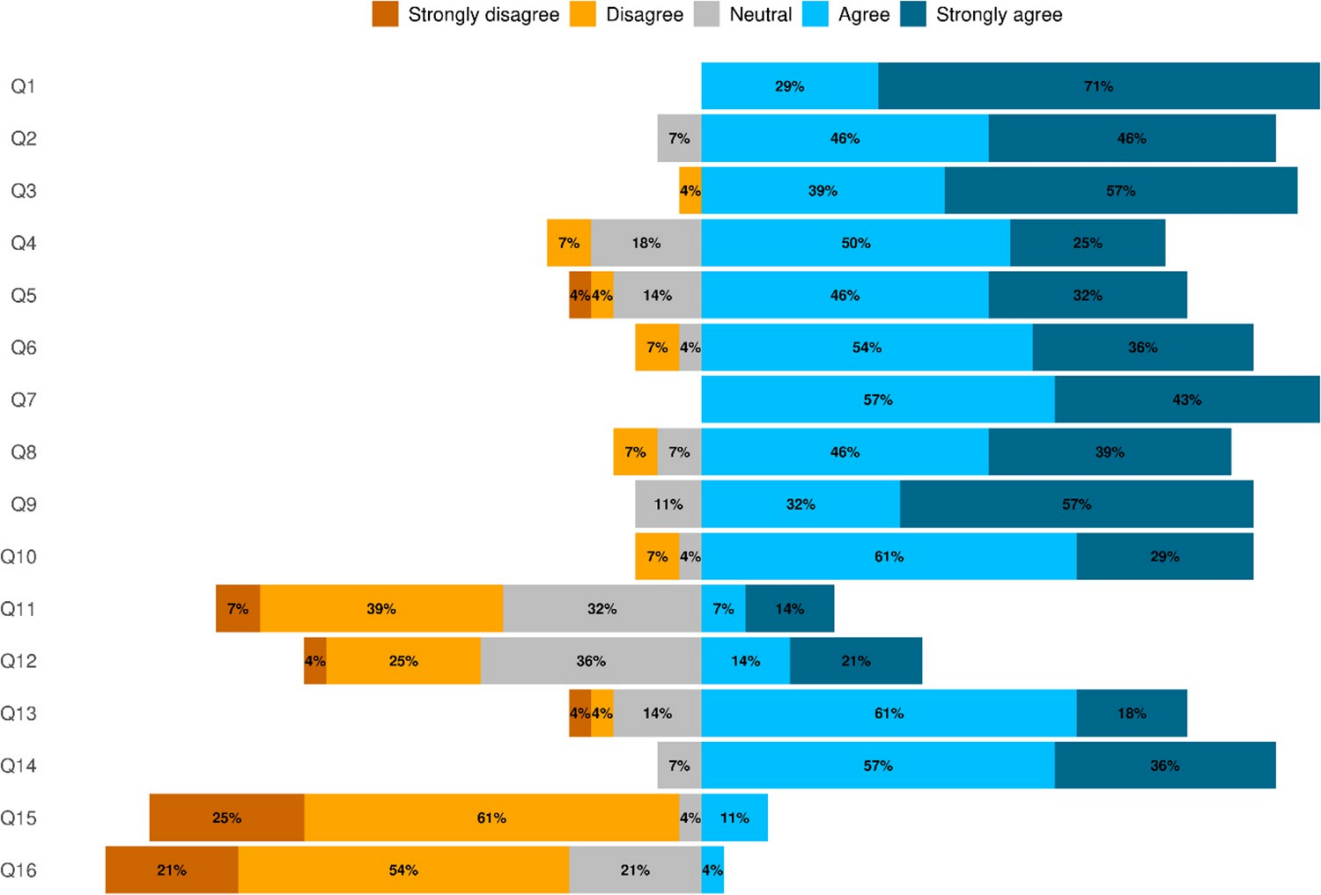
Summary results from the 19-question questionnaire used to query 27 bear management and research biologists from federal (non-National Park Service), state and provincial agencies, and universities across North America.

All 27 non-NPS respondents agreed that baiting bears with large quantities (e.g., 100s of lbs.) of processed foods as allowed under Alaska state law was functionally equivalent to feeding bears (Fig 2). Of the non-NPS respondents, 92.59% thought bears would defend a bait station provisioned with processed foods in a manner equivalent to how that bear would defend a carcass (the remaining two respondents were neutral). Of the 27 non-NPS respondents, 7.40% thought bear baiting with large quantities of processed foods would not lead to bears associating food with humans. While 11.11% of non-NPS respondents thought a ¼ mile buffer around trails would resolve the public safety concerns of a bear defending a bait station with large quantities of processed foods, 3.70% of the non-NPS respondents (i.e., a single respondent) thought a 1-mile buffer around dwellings would resolve the public safety concerns of bears associating food with people. All of the 27 non-NPS respondents unanimously agreed that baiting with large quantities of processed foods would alter natural bear behavior and could impact the broader ecosystem, including non-target species.

Regarding baiting with large quantities of natural foods (e.g., ungulate carcasses, salmon, berries), 85.19% of the non-NPS respondents agreed this provisioning treatment is functionally equivalent to feeding bears, whereas 7.40% of non-NPS respondents disagreed and 7.40% of non-NPS respondents were neutral. Of the 27 non-NPS respondents, 88.88% agreed that bears would defend a bait station provisioned with natural foods in a manner equivalent to how a bear would defend a carcass. Similarly, 88.88% of non-NPS respondents agreed that baiting with natural foods may alter natural bear behavior. However, there was less agreement among non-NPS respondents regarding whether natural food items might lead to human food-conditioned bears (strongly disagree 7.40%, disagree 40.74%, neutral 33.33%, agree 7.04%, strongly agree 14.81%). Additionally, there was no consensus among non-NPS respondents as to whether using large quantities of natural foods may contribute to an increase in the killing of bears in defense of life or property (strongly disagree 3.70%, disagree 25.93% neutral 37.04%, agree 14.85%, strongly agree 18.52%). However, 77.78% of non-NPS respondents agreed that baiting with natural foods may impact bear behavior beyond the immediate baiting season and 92.59% of non-NPS respondents agreed that baiting with natural foods may impact other components of the ecosystem beyond bears (i.e., non-target species). In regard to public safety concerns, 25.93% of non-NPS respondents strongly disagreed and 62.96% disagreed that that the ¼-mile buffer around trails and roads was sufficient for resolving concerns. Similarly, 74.07% of non-NPS respondents disagreed that the 1-mile buffer around cabins or other dwellings resolves public safety and property damage concerns.

## Discussion

Managing for multiple legal mandates within and across agencies is complex in boundary areas and even more so when jurisdictions overlap. In 2015 and 2020, the National Park Service embarked on long and expensive rule-making processes to determine whether or not to allow bear baiting on National Preserves in Alaska. In each case, definitive information on the potential risks to humans and wildlife and the efficacy of viable mitigations was lacking. In cases in which empirical data are not available, use of expert opinion is a viable method to inform decision making [14]. Thus, our intent with this study is to inform future decisions with expert opinion. The NPS-administered questionnaire was informed by ~280 collective years of natural resource management experience and the non-NPS questionnaire was informed by ~700 years of collective bear management and research experience.

The consensus of expert opinion is that bear baiting is functionally equivalent to feeding bears, bears are likely to defend a bait station in a manner similar to how they would defend a carcass, and baiting can lead to human food-conditioning in bears. Further, baiting increases the likelihood that bears will be killed in defense of life and property [11–14] and baiting alters natural behaviors and ecological processes (S2 Table). The aforementioned concerns are noted in the open-ended comments from the respondents (S2 Table: column W). For example, below are a few representative quotes from the NPS and non-NPS respondents:

“*The core issue surrounding most human-bear conflicts is human-provisioned food resources. Bear baiting introduces increased opportunities for bears to alter their natural behavior in relation to human-provisioned food. Bears are extremely food motivated, and quickly learn to associate food with people, or human occupied areas. Bears that become conditioned to human-provisioned foods tend to lose their fear of humans, and may become more bold in their efforts to obtain food from people. Both natural and unnatural (e.g., dogfood, human foods, garbage) human-provisioned foods have been known to increase negative interactions between bears and people.”*

     -*Non-NPS Respondent 13*

“In areas of grizzly bear habitat in [State 1], [State 2], [State 3], and [State 4] the National Forests have food storage order to limit access to human foods by bears and other wildlife. Allowing baiting with human foods seems counterproductive to those efforts particularly when attempting to recover bears and limit conflict behavior in bears.”

     -*Non-NPS Respondent 6*

“…in my view, baiting with human-derived foods is in contradiction to most of what we try to do in bear management—i.e., keep bears away from human foods… But, a single moose carcass would, in my opinion, be much preferable to human-derived foods.

     -*Non-NPS Respondent 12*

“[State 5] has always allowed baiting by hunters (with increased restrictions on the number and types of baits). We have seen no correspondence between collared bears known to use baits and their tendency to use human sources of food during the non-hunting season. In fact, if anything, the association goes in the opposite direction: those bears that tend toward conflicts, and those bears trapped multiple times during the summer are more apt to be attracted to bait used by hunters, and thus killed (eliminated from population).”

     -*Non-NPS Respondent 18*

“We really know very little about how baiting affects bear-human interactions. The bait certainly can alter the physiology of bears and affect population demographics, but whether bears associate bait with humans is simply unknown. Therefore, it is difficult to say whether bait will affect non-hunting mortalities, conflict complaints, etc.

     -*Non-NPS Respondent 21*

"I do think bear baiting has the potential to create a risk to public safety. The risk level depends on how many people live in the vicinity of the bear baiting. In very remote areas of the state, the risk of a bear becoming food conditioned from bear baiting and then looking for food from people in villages or homesteads would be lower than in areas where more people live. If hunters are baiting bears near where people live and recreate I would say the risk to public safety could be as high as 5." "Bear baiting can absolutely change the natural behavior of bears and other wildlife by intentionally feeding them anthropogenic food, thus teaching them to search for and obtain such foods. When bears are baited by human foods, bear baiting can create the exact problem that bear managers across the world are continually trying to prevent—a food conditioned bear that is actively searching for food from people."

     -* NPS Respondent 3*

"The attractants used in baiting have the ability to alter movements and food habits of bears in the area. This artificial source can concentrate bears in areas where human presence is likely higher (moving/establishing bait stations requires relatively easy human access) which increases the likelihood of human-bear interactions. More interactions with bears that are essentially being food conditioned will likely lead to greater conflicts."

     *- NPS Respondent 11*

Another point of concern regarding bear baiting identified by the NPS questionnaire is that current mitigation measures are inadequate for resolving public safety concerns or property damage concerns due to bears associating food with people (S2 Table). For example, although bear baiting relies largely on ease of access for establishing and maintaining bait stations, NPS land access in Alaska is challenging or limited due to few public roads [15]. In fact, the only National Preserve in Alaska with substantial public road access is Wrangell–St. Elias National Preserve. Though few data are available, 73% of bear bait stations located by NPS personnel in Wrangell-St. Elias National Park and Preserve from 1996–2004 were not in compliance with state or federal regulations (NPS Internal Records). Specifically, common state violations included: 70% within ¼ mile of the McCarthy Road, 24% failure to remove bait and/or bear baiting equipment after the season closed, 6% within a mile of house or other permanent dwelling. Common federal violations included 59% illegal tree cutting, 25% illegal off-road-vehicle use, 8% sport hunting within a prohibited area (i.e., national park), and 8% improperly disposing of refuse (NPS Report 2004). In short, the mitigating strategies in place fell short of resolving public safety risks or concerns for property damage during that time period (1996–2004).

The perspectives above are consistent with the State of Alaska’s management plan for Denali State Park, which is managed by the State of Alaska Department of Natural Resources and, similar to National Preserves, has a broader mission that considers the visitor experience and safety of all visitors. Thus, State Parks officials strive to balance a variety of uses. As one example, the [16] Denali State Park Management Plan discusses concerns that bear baiting ‘‘teaches bears to associate humans with food sources” and states that bear baiting is in direct conflict with recreational, non-hunting uses of the park. The plan further notes that bear baiting has ‘‘the potential for creating serious human-bear conflicts, by encouraging bears to associate with campgrounds and other human congregation points with food sources.”

If an agency’s primary mandate is to manage harvest and provide for hunter opportunity, the authorization of bear baiting is consistent with that agency’s mission. However, for agencies with a broader mission that includes public safety and maintaining natural ecosystems, natural wildlife behaviors, and natural ecological processes, the authorization of bear baiting is far more complex due to the risks and impacts identified in this study. While the National Park Service does allow and permit high risk activities such as mountain climbing, those engaging in these activities have the opportunity to voluntarily assume risk. In contrast, the risks posed by bear baiting extend more broadly to anyone using the area in proximity of the bait station or any area a food-conditioned bear may roam.

Based on the results of this study, it is broadly recognized in the field of bear management that bear baiting, as practiced in Alaska, poses a human safety risk both immediately (at a bait station) and as a consequence of human food-conditioning of bears. Further, baiting has the potential to contribute to the loss of life of individual bears (a resource in and of themselves) through non-harvest mortality and may contribute to property damage by baited bears. In addition to public safety concerns and resource conservation, bear baiting impacts ecosystem processes at both broad scales (e.g., nutrient subsidies for bears and non-target species) and small scales (e.g., within the GI tract of individual animals [5, 9, 10]),. Not only are the risks to the above values knowable and foreseeable, these risks are preventable through the thoughtful management of permissible activities within National Preserves.

## Supporting information

S1 Table 5-question questionnaire used to query 14 natural resource and research technical experts from the National Park Service including each anonymous respondent’s answer to each question. (XLSX)

S2 Table 19-question questionnaire used to query 27 bear management and research biologists from federal (non-National Park Service), state and provincial agencies, and universities across North America including each anonymous respondent’s answer to each question. (XLSX)
